# Axial spondyloarthritis in patients with recurrent fever attacks: data from the AIDA network registry for undifferentiated autoInflammatory diseases (USAIDs)

**DOI:** 10.3389/fmed.2023.1195995

**Published:** 2023-05-30

**Authors:** Antonio Vitale, Valeria Caggiano, Isabel Silva, Daniel G. Oliveira, Piero Ruscitti, Francesco Ciccia, Ibrahim Vasi, Abdurrahman Tufan, Giuseppe Lopalco, Ibrahim A. AlMaghlouth, Jurgen Sota, Ewa Wiesik-Szewczyk, Carla Gaggiano, Henrique Ayres Mayrink Giardini, Veronica Spedicato, Gaafar Ragab, Florenzo Iannone, Alberto Balistreri, Micol Frassi, José Hernández-Rodríguez, Claudia Fabiani, Paolo Falsetti, Nunzia Di Meglio, Bruno Frediani, Maria Antonietta Mazzei, Donato Rigante, Raquel Faria, Luca Cantarini

**Affiliations:** ^1^Department of Medical Sciences, Surgery and Neurosciences, Research Center of Systemic Autoinflammatory Diseases and Behçet’s Disease Clinic, University of Siena, Siena, Italy; ^2^Azienda Ospedaliero-Universitaria Senese [European Reference Network (ERN) for Rare Immunodeficiency, Autoinflammatory and Autoimmune Diseases (RITA) Center] Siena, Siena, Italy; ^3^Medicine Department, Centro Hospitalar Universitário de Santo António, Porto, Portugal; ^4^UMIB—Unit for Multidisciplinary Research in Biomedicine, ICBAS School of Medicine and Biomedical Sciences, University of Porto, Porto, Portugal; ^5^Instituto de Investigação e Inovação em Saúde, Universidade do Porto, Porto, Portugal; ^6^Rheumatology Unit, Department of Biotechnological and Applied Clinical Sciences, University of L’Aquila, L’Aquila, Italy; ^7^Department of Precision Medicine, Università Degli Studi Della Campania Luigi Vanvitelli, Naples, Italy; ^8^Division of Rheumatology, Department of Internal Medicine, Gazi University Faculty of Medicine, Ankara, Türkiye; ^9^Rheumatology Unit, Department of Emergency and Organ Transplantation, University of Bari, Bari, Italy; ^10^Rheumatology Unit, Department of Medicine, King Saud University, Riyadh, Saudi Arabia; ^11^Department of Internal Medicine, Pneumonology, Allergology and Clinical Immunology, Central Clinical Hospital of the Ministry of National Defense, Military Institute of Medicine, National Research Institute, Warsaw, Poland; ^12^Rheumatology Division, Faculdade de Medicina, Hospital das Clínicas da Faculdade de Medicina da Universidade de São Paulo (HCFMUSP), Universidade de São Paulo, São Paulo, Brazil; ^13^Internal Medicine Department, Rheumatology and Clinical Immunology Unit, Faculty of Medicine, Cairo University, Giza, Egypt; ^14^Faculty of Medicine, Newgiza University, Giza, Egypt; ^15^Bioengineering and Biomedical Data Science Lab, Department of Medical Biotechnologies, University of Siena, Siena, Italy; ^16^Rheumatology and Clinical Immunology, Spedali Civili and Department of Clinical and Experimental Sciences, University of Brescia [European Reference Network (ERN) for Rare Immunodeficiency, Autoinflammatory and Autoimmune Diseases (RITA) Center], Brescia, Italy; ^17^Vasculitis Research Unit and Autoinflammatory Diseases Clinical Unit, Department of Autoimmune Diseases, Hospital Clinic of Barcelona, IDIBAPS, University of Barcelona [European Reference Network (ERN) for Rare Immunodeficiency, Autoinflammatory and Autoimmune Diseases (RITA) Center], Barcelona, Spain; ^18^Ophthalmology Unit, Department of Medicine, Surgery and Neurosciences, University of Siena, Siena, Italy; ^19^Unit of Diagnostic Imaging, Department of Medical, Surgical and Neuro Sciences and of Radiological Sciences, University of Siena, Azienda Ospedaliero-Universitaria Senese, Siena, Italy; ^20^Department of Life Sciences and Public Health, Fondazione Policlinico Universitario A. Gemelli IRCCS, Rome, Italy; ^21^Rare Diseases and Periodic Fevers Research Centre, Università Cattolica Sacro Cuore, Rome, Italy; ^22^Unidade de Imunologia ClínicaCentro Hospitalar Universitário de Santo António Porto [European Reference Network (ERN) for Rare Immunodeficiency, Autoinflammatory and Autoimmune Diseases (RITA) Center], Porto, Portugal

**Keywords:** arthritis, autoinflammatory diseases, diagnosis, outcome, SpA, treatment

## Abstract

**Beckground:**

Despite the recent advances in the field of autoinflammatory diseases, most patients with recurrent fever episodes do not have any defined diagnosis. The present study aims at describing a cohort of patients suffering from apparently unexplained recurrent fever, in whom non-radiographic axial spondylarthritis (SpA) represented the unique diagnosis identified after a complete clinical and radiologic assessment.

**Materials and methods:**

Patients’ data were obtained from the international registry on Undifferentiated Systemic AutoInflammatory Diseases (USAIDs) developed by the AutoInflammatory Disease Alliance (AIDA) network.

**Results:**

A total of 54 patients with recurrent fever episodes were also affected by non-radiographic axial SpA according to the international classification criteria. SpA was diagnosed after the start of fever episodes in all cases; the mean age at the diagnosis of axial SpA was 39.9 ± 14.8 years with a diagnostic delay of 9.3 years. The highest body temperature reached during flares was 42°C, with a mean temperature of 38.8 ± 1.1°C. The most frequent manifestations associated to fever were: arthralgia in 33 (61.1%) cases, myalgia in 24 (44.4%) cases, arthritis in 22 (40.7%) cases, headache in 15 (27.8%) cases, diarrhea in 14 (25.9%) cases, abdominal pain in 13 (24.1%) cases, and skin rash in 12 (22.1%) cases. Twenty-four (44.4%) patients have taken daily or on-demand non-steroidal anti-inflammatory drugs (NSAIDs) and 31 (57.4%) patients have been treated with daily or on demand oral glucocorticoids. Colchicine was used in 28 (51.8%) patients, while other conventional disease modifying anti-rheumatic drugs (cDMARDs) were employed in 28 (51.8%) patients. Forty (74.1%) patients underwent anti-tumor necrosis factor (TNF) agents and 11 (20.4%) were treated with interleukin (IL)-1 inhibitors. The response to TNF inhibitors on recurrent fever episodes appeared more effective than that observed with anti-IL-1 agents; colchicine and other cDMARDs were more useful when combined with biotechnological agents.

**Conclusion:**

Signs and symptoms referring to axial SpA should be inquired in patients with apparently unexplained recurrent fever episodes. The specific treatment for axial SpA may lead to a remarkable improvement in the severity and/or frequency of fever episodes in patients with unexplained fevers and concomitant axial SpA.

## 1. Introduction

Fever is an active, yet unspecific response of the innate immune system aimed at neutralizing the harmful cause leading to cytokines release. In addition to infectious and neoplastic diseases, recurrent fever episodes may associate to autoimmune or autoinflammatory disorders, such as hereditary periodic fever syndromes, Still’s disease, Schnitzler syndrome, or PFAPA (Periodic Fever, Aphthous stomatitis, Pharyngitis and cervical Adenitis) syndrome (1, 2). In addition to fever, autoinflammatory diseases manifest with a protean spectrum of inflammatory manifestations especially involving joints, the eye, the skin, and serosal membranes (3, 4).

Patients suffering from recurrent fever episodes need an accurate differential diagnosis including all possible causes of inflammation. This process is usually not straightforward and requires careful evaluation of the clinical history and manifestations, followed by a full history-driven laboratory and radiological workup (2). Among the large number of systemic diseases capable of manifesting with systemic inflammation and fever, arthritic conditions should also be considered (5, 6). In this perspective, the presence of fever has been also reported in patients with spondylarthritis (SpA), while Byun et al. have recently shown that fever may account for the initial disease manifestation in various SpA subtypes (7).

Axial SpA is the second most prevalent form of chronic inflammatory arthritis, with an estimated prevalence of 0.5%–1.5% in the Caucasian population (8, 9). It is characterized by inflammation of the spine and sacroiliac joints, with or without peripheral articular involvement. Extra-articular sites may be also involved by inflammation, with the eye, the gut, and the skin especially interested (10). The role of innate immunity in the development of axial SpA has been recently put under the magnifying glass and an autoinflammatory pathogenesis of the disease has consequently been supposed. In this regard, the role of the NLRP3 inflammasome, an intracellular multiprotein complex primarily involved in many autoinflammatory disorders, has been increasingly implicated in patients with SpA. Special interest has been raised by the identification of a specific pattern of SpA associated with gene mutations capable of inducing inflammasome dysfunction (11, 12). Among others, mutations affecting the *MEFV* gene, which is responsible for familial Mediterranean fever (FMF) and encodes the protein pyrin, an essential regulator of the NLRP3 inflammasome, have been associated to an increased frequency of SpA, disregarding the HLA-B27 haplotype (13, 14).

Based on this preliminary evidence, we inquired the presence of inflammatory low back pain and/or axial SpA signs at physical examination among patients with recurrent fever episodes. Tehus, the present study aims at describing a cohort of patients suffering from apparently unexplained recurrent fever, in whom axial SpA was diagnosed according to the Assessment of SpondyloArthritis international Society (ASAS) criteria during the diagnostic workup (15).

## 2. Materials and methods

The patients were consecutively identified and enrolled from April 2021 to November 2022. Data were collected in the International AIDA (AutoInflammatory Disease Alliance) network registry for Undifferentiated Systemic AutoInflammatory Diseases (USAIDs) (16). This registry represents an observational study collecting data obtained from patients managed and treated according to the best standard of care, based on patients’ history, and tailored on clinical and laboratory manifestations.

All patients were newly diagnosed with non-radiographic axial SpA during the diagnostic workflow related to undiagnosed fever (at least one febrile episode was evaluated and confirmed by physicians). ASAS criteria (imaging arm) were fulfilled in all cases (15). In particular, all subjects referred inflammatory low back pain and other extra-articular affections ascribable to SpA. A subsequent magnetic resonance imaging of sacroiliac joints confirmed the presence of radiologic signs of axial SpA (15). Enteropathic SpA patients were diagnosed when an inflammatory bowel disease coexisted with arthritis. Reactive SpA was excluded, as no patients presented gastrointestinal and/or urinary infections in the 6 months prior of the onset of symptoms. Patients <16 year-old were also included.

Disease duration was defined as the time between the onset of articular symptoms and the diagnosis of axial SpA. Infections, autoimmune and neoplastic diseases were excluded in all patients after a complete laboratory and instrumental assessment. Next generation sequencing was performed to search for any genetic variant linked to the currently known monogenic autoinflammatory diseases. Among others, *NLRP3*, *TNFRSF1A*, *MEFV*, *MVK* and *NOD2* genes were investigated in all cases. Patients did not have to fulfil any criteria for genetically determined FMF or any multifactorial autoinflammatory diseases, including PFAPA syndrome, Behçet’s disease, Schnitzler syndrome, Castleman disease, Still’s disease (17–27). Laboratory findings, including C-reactive protein (CRP), erythrocyte sedimentation rate (ESR), and human leukocyte antigen (HLA)-B27 test, were collected at the time of diagnosis and at the last assessment.

The objective of this paper is to describe a large cohort of patients diagnosed with axial SpA during the diagnostic process related to otherwise unexplained recurrent fever episodes.

The aim of this paper is to assess the demographic, clinical, laboratory and therapeutic features of patients with axial SpA and recurrent fever episodes. In particular, clinical manifestations accompanying fever and response to different treatment approaches were investigated.

Regarding treatment outcomes, *complete response* was defined as the resolution of all disease-related clinical manifestations, with decrease to normal values of all laboratory inflammatory parameters. *Partial response* was defined as persistence of clinical manifestations with remarkable decrease in their severity, as reported by patients, with inflammatory laboratory parameters normalized or only slightly increased. *Failure* was meant as persistence of fever-associated clinical manifestations and/or no decrease of laboratory inflammatory markers. When available, axial SpA clinical activity was assessed with Bath Ankylosing Spondylitis Funcional Index (BASFI) and the Bath Ankylosing Spondylitis Disease Activity Index (BASDAI) among patients treated with TNF inhibitors (28, 29). The terms *adverse event* referred to any untoward medical occurrence observed after the exposure to each treatment taken by the patients due to ax-SpA and not necessarily caused by the treatment.

All patients or parents or legal guardian signed the informed consent to participate to this project. The study has been approved by the Ethics Committee of Azienda Ospedaliero Universitaria Senese, Siena, Italy (AIDA Project; Ref. N. 14’951) as part of the AIDA Program. The study protocol conformed to the tenets of the Declaration of Helsinki.

Regarding statistical computations, descriptive statistics included mean, standard deviation (SD), mode, range, median and interquartile range (IQR), as required. Data distribution was evaluated by the Shapiro–Wilk test. Pairwise computations of quantitative data were performed with student *t*-test or Wilcoxon test, according to data distribution; pairwise computations of qualitative data were performed by Fisher exact test with 2 × 2 contingency tables. Significance level was set at 95% (*p*-value <0.05); *p*-values were two-tailed. Statistical analysis was performed through the Stata 17/MP2 software.

## 3. Results

A total of 54 patients affected by non-radiographic axial SpA referred to our outclinic services because of recurrent fever episodes, between April 2021 and November 2022. None of the patients suffered from reactive axial SpA; all patients fulfilled the ASAS criteria (15).

### 3.1. Demographic features

The enrolled patients were aged 41.8 ± 13.3 years (median: 41.3 years; mode: 50 50.5 54.4 years; range: 20.3–68.4 years), and were mainly females (*n* = 36, 66.7%). All patients were Caucasian. Recurrent fever episodes firstly occurred during childhood or adolescence (<16 years) in 14 (25.9%) patients, with a mean age at onset of 10.6 ± 4.6 years (median: 12 years; mode: 12 years; range: 0.8–15 years).

SpA was diagnosed after the start of fever episodes in all cases; the mean age at the diagnosis of axial SpA (median: 42 years; mode: 42 50 50.5 55.6 years; range: 13.8–68.4 years) with a diagnostic delay of 9.3 years from the start of articular symptoms and a disease duration of 9.7 ± 10.7 years. Five (9.3%) patients carried HLA-B27. Thirty-eight (70.4%) patients had at least one comorbidity, as described in Table 1.

**Table 1:** List of comorbidities observed in the cohort of patients enrolled in present study; percentages included in the table refer to the total number of patients with at least one comorbidity.

Comorbidity	*n* (%)
Chronic gastritis	7 (18.4)
Hypertension	4 (10.5)
Bronchial asthma	4 (10.5)
Autoimmune thyroiditis	3 (7.9)
Diverticulosis	3 (7.9)
Steatohepatitis	2 (5.3)
Atopic eczema	2 (5.3)
Allergic rhinitis	2 (5.3)
Favism	2 (5.3)
Healthy carrier of thalassemia	2 (5.3)
Coeliac disease	2 (5.3)
Osteoporosis	2 (5.3)
Adenomyosis	2 (5.3)
Hypercholesterolaemia	1 (2.6)
Lymphangioma	1 (2.6)
Pulmonary emphysema	1 (2.6)
Von Willebrand disease type 1	1 (2.6)
Hepatic angioma	1 (2.6)
Vitiligo	1 (2.6)
Primary biliary cholangitis	1 (2.6)
Caroli disease	1 (2.6)
Papillary thyroid carcinoma	1 (2.6)
Basal-cell carcinoma of nose	1 (2.6)
Childhood rheumatic disease	1 (2.6)
Endometriosis	1 (2.6)
Arnold chiari malformation	1 (2.6)
Sensorineural hearing loss	1 (2.6)
Autoimmune pancreatitis	1 (2.6)
Leukocytoclastic vasculitis	1 (2.6)
Disorder of adrenal gland	1 (2.6)
Meniere’s disease	1 (2.6)
Lichen sclerosus et atrophicus	1 (2.6)
Thrombophlebitis	1 (2.6)
Erythema nodosum	1 (2.6)
Ocular hypertension	1 (2.6)
Chronic renal failure	1 (2.6)
Epilepsy	1 (2.6)
Recurrent perimyocarditis	1 (2.6)
IgA Nephropathy	1 (2.6)
Urticarial rash	1 (2.6)
Angioedema	1 (2.6)
Cerebrovascular disease	1 (2.6)
Gout	1 (2.6)

A genetic assessment was performed to rule out any variant affecting genes responsible for known monogenic autoinflammatory diseases. No pathogenic or likely pathogenic mutation was identified.

### 3.2. Features of fever attacks and triggers

**Figure 1:**
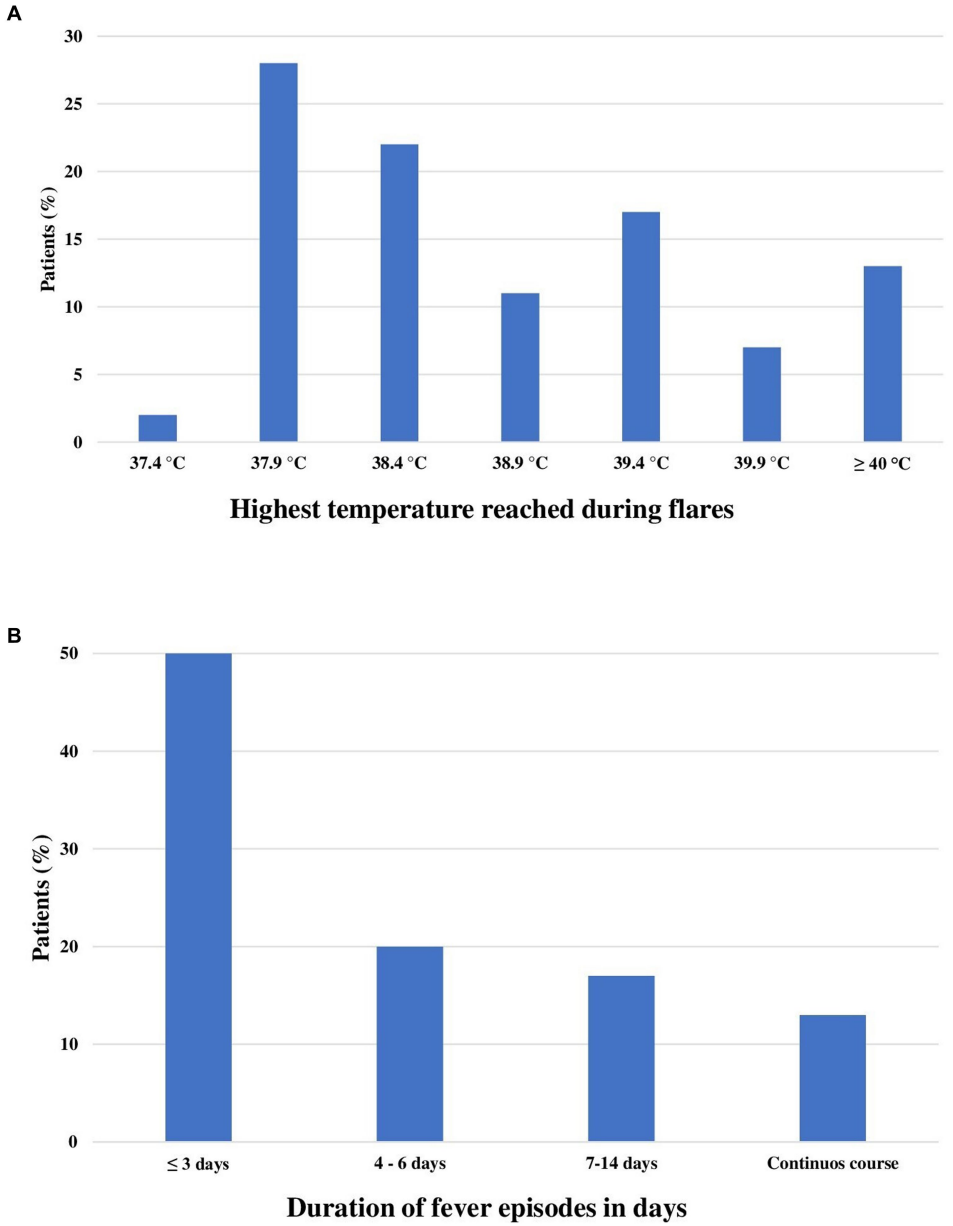
Bar charts describe the highest temperature observed during flares **(A)** and the mean duration of fever episodes **(B)** among the 54 patients enrolled.

The highest body temperature reached during flares was 42°C, with a mean temperature of 38.8 ± 1.1°C in the whole cohort. The median frequency of fever episodes at the time of the enrolment was 6 episodes per year. Figure 1A illustrates the distribution of patients according to the highest temperature reached during flares and the duration of fever episodes in days. Figure 1B provides information about the duration of fever episodes.

**Figure 2:**
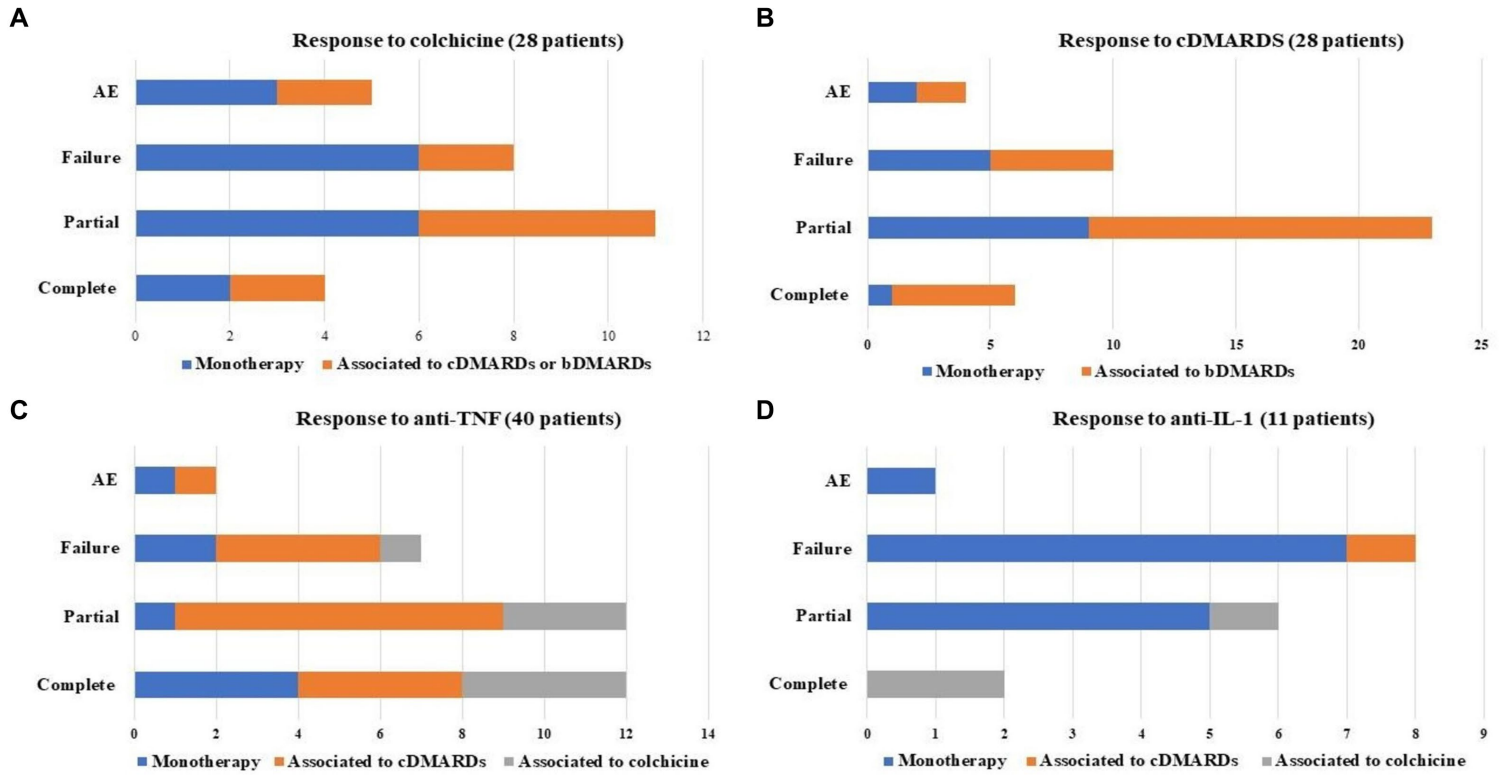
Response to different treatments performed by patients included in the study; monotherapy and combination treatment with conventional or biotechnological disease modifying anti-rheumatic drugs (cDMARDs and bDMARDs, respectively) or colchicine were pointed out. The figure specifically describes treatment with colchicine **(A)**, cDMARDs **(B)**, anti-tumor necrosis factor (TNF) **(C)**, and anti-interleukin (IL)-1 **(D)**.

The clinical manifestations accompanying fever are described in Table 2. Peripheral arthritis was observed in 22 patients in the form of monoarthritis (*n* = 3), oligoarthritis (*n* = 7), and polyarthritis (*n* = 12). Intraocular inflammation was reported in 7 (13%) patients; 5 (9.3%) patients presented psoriasis and none of the other patients referred a positive family history for psoriasis; 3 (5.5%) patients suffered from intestinal inflammation. In detail, 2 patients were affected by undifferentiated intestinal inflammation and the last one suffered from Crohn’s disease.

**Table 2:** Frequency of most common clinical manifestations reported during fever episodes.

Clinical manifestations	Number of patients (%)
Arthralgia	33 (61.1)
Myalgia	24 (44.4)
Arthritis	22 (40.7)
Headache	15 (27.8)
Diarrhea	14 (25.9)
Abdominal pain	13 (24.1)
Skin rash	12 (22.2)
Oral aphthosis	10 (18.5)
Lymphoadenitis	10 (18.5)
Pharyngitis	9 (16.7)
Thoracic pain	7 (12.9)
Genital aphthosis	3 (5.5)
Splenomegaly	3 (5.5)
Vomiting	3 (5.5)

Triggers inducing relapses were reported in 17 (31.5%) patients at the time of the diagnosis: psychological stress in 11 patients, physical activity in 8 subjects, generalised cold exposure in 7 patients, menstrual phase in 7 females, and generalised exposure to warm temperatures in 4 patients. One patient relapsed after SARS-CoV-2 vaccination.

Laboratory investigations recorded during an intercritic period before starting any specific treatment with conventional and or biotechnological disease modifying anti-rheumatic drugs (cDMARDs and/or bDMARDs) showed increased inflammatory markers (CRP and/or ESR) in 30 (55.5%) patients and leucocytosis in 9 (16.6%) subjects.

### 3.3. Therapeutic approaches

Regarding treatment, 24 (44.4%) patients have taken daily or on-demand non-steroidal anti-inflammatory drugs (NSAIDs) and 31 (57.4%) patients have been treated with daily or on demand oral glucocorticoids. Therapy with colchicine up to 1 mg/day was attempted in 28 (51.8%) patients. Colchicine was discontinued in 17 (60.7%) patients due to: adverse events (*n* = 5, 29.4%), especially diarrhoea (*n* = 4), abdominal and/or pelvic pain (*n* = 3), nausea (*n* = 2) and exacerbation of hemorrhoids inflammation (*n* = 1); lack of efficacy (*n* = 5, 29.4%); and loss of efficacy (*n* = 5, 29.4%). Two further patients (11.8%) stopped colchicine after the introduction of the biotechnological treatment. Twenty-eight (51.9%) patients were treated with cDMARDs as follow: 16 (29.6%) with methotrexate, 15 (27.8%) with sulfasalazine, 6 (11.1%) with hydroxychloroquine, 4 (7.4%) patients with leflunomide, 1 (1.8%) with mesalazine and 1 (1.8%) with azathioprine. Figures 2A,B provides details about the response to colchicine and to cDMARDs. Four patients discontinued cDMARDs due to adverse events as follow: localized skin reaction to methotrexate (*n* = 1); severe nausea after sulfasalazine introduction (*n* = 1); epigastric pain during sulfasalazine (*n* = 1); diarrhea during leflunomide treatment (*n* = 1).

Adalimumab was the most widely biotechnological agent employed (*n* = 28, 51.8%), followed by anakinra and canakinumab used in 9 (16.6%) and 8 (14.8%) patients respectively; intravenous infliximab was employed in 5 (9.2%) patients, etanercept in 4 (7.4%) patients, secukinumab in 3 (5.6%) cases, golimumab in 2 cases (3.7%), tocilizumab in 1 subject (1.8%), and certolizumab pegol in 1 subject (1.8%). The therapy with the Janus kinase (JAK) inhibitor Tofacitinib was used in 2 (3.7%) patients.

**Figure 3:**
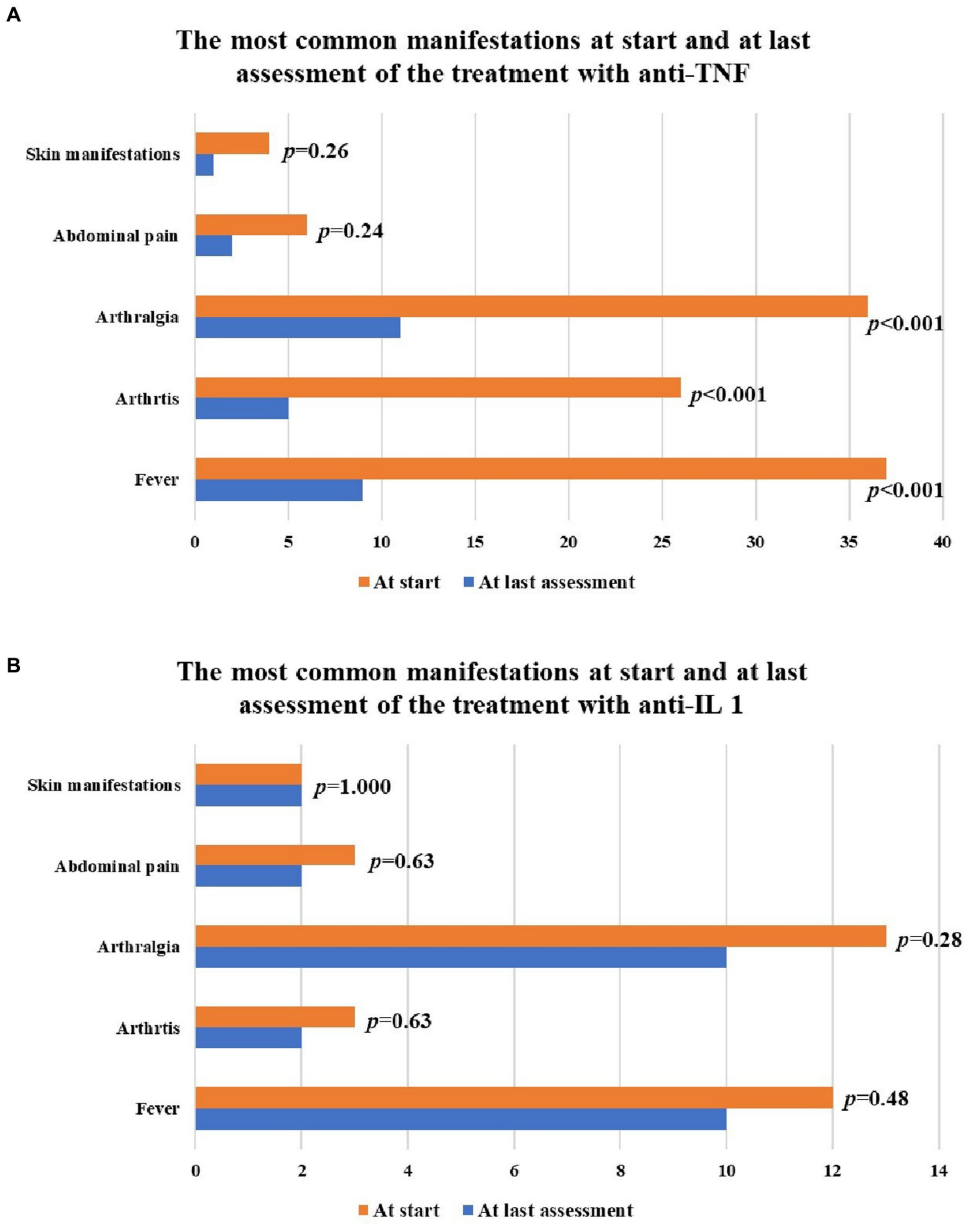
Bar charts describe the frequency of the main clinical manifestations observed during inflammatory attacks at the start and at the last assessment while on treatment with anti-tumor necrosis factor (TNF) agents **(A)** and interleukin (IL)-1 inhibitors **(B)**. Skin manifestations observed among patients treated with TNF inhibitors consisted of urticarial skin rash in 2 patients, pustular rash in one patient, erythematous skin rash in one patient; the latter one persisted at the last assessment. Skin manifestations observed among patients treated with IL-1 inhibitors consisted of erythematous skin rash in one patient and maculo-papular skin rash in a second patient. None of them resolved during IL-1 inhibition.

A total of 40 (74.1%) patients performed a treatment with anti-TNF agents; treatment duration was 7.0 ± 6.6 months (median value: 3 months). A minimum follow-up of three months was available in 32 patients. Twelve (30%) patients were treated with glucocorticoids at the dosage of 9.6 ± 6.1 mg/day (prednisone or equivalent) at the start of anti-TNF treatment; this dosage decreased to 6.25 ± 2.5 mg/day at the last assessment (*p* = 0.01). Figure 2C highlights the response to anti-TNF agents. Generalised skin reactions accounted for the adverse events leading to stop the anti-TNF treatment in two cases (treated with adalimumab and infliximab). Figure 3A provides the frequency of fever, arthritis, arthralgia, skin manifestations and abdominal pain at the start and at the last visit while on treatment with TNF inhibitors (last visit available in 32 patients). The BASDAI and BASFI at the beginning of anti-TNF therapy were 6.2 ± 2.4 and 4.3 ± 2.0, respectively; at the last assessment they were 4.2 ± 2.1 and 3.0 ± 2.5. The decrease of BASDAI (*p* = 0.7) and BASFI (*p* = 0.24) scores did not reach statistical significance.

Thirty out of 40 (75%) patients treated with anti-TNF agents showed increased ESR and/or CRP at the start of the treatment; the number of patients with increased inflammatory markers was 17 out of 32 (53.1%) patients at the last assessment (*p* = 0.09). The median CRP was 0.48 (IQR = 2.46) mg/dL at the beginning of anti-TNF therapy and 0.28 (IQR = 0.51) mg/dL at the last follow-up (*p* = 0.06); the median ESR was 20 (IQR = 26.75) mm/h at the start of treatment and 10 (IQR = 10.5) mm/h at last assessment (*p* = 0.01).

As a whole, 11 (20.4%) patients were treated with IL-1 antagonists, corresponding to 17 treatment courses. The median glucocorticoids dosage was 9.2 ± 5.9 mg/day (prednisone or equivalent) at the start of anti-IL-1 treatment and 9.2 ± 5.9 mg/day at the last assessment (*p* = 1.000). Figure 2D describes the response to IL-1 antagonists, while Figure 3B provides the frequency of fever, arthritis, arthralgia, skin manifestations and abdominal pain at the start of IL-1 antagonists and the last visit. One adverse event leading to anakinra discontinuation consisted of a generalised skin reaction.

Secukinumab was used as monotherapy in 3 cases, leading to complete response in 1 patient, failure in the second patient, discontinuation due to angioedema in the third patient. The patient treated with tocilizumab underwent complete response. The 2 patients treated with tofacitinib showed complete response in one case and partial response in the second case.

## 4. Discussion

Fever is one of the most common signs that physicians meet in clinical practice. It is generally associated to a widespread spectrum of diseases, including infections, neoplastic pathologies, autoimmune diseases, and autoinflammatory disorders. Nevertheless, some patients do not have any defined diagnosis despite the full laboratory and radiological assessments, turning these clinical cases into a diagnostic and therapeutic conundrum. The recent advances in the field of autoinflammatory diseases have increasingly opened the door to the diagnosis in a greater number of patients, but at least 40% of cases with an autoinflammatory picture do not fall into any of the currently known diseases (30). This makes further efforts necessary to better understand the nature and the correct treatment of unexplained periodic fever episodes. In this context, the AutoInflammatory Disease Alliance (AIDA) project has recently supported the development of an international registry dedicated to undifferentiated autoinflammatory conditions, paving the way to suitable clinical research possibly leading to the identification of new clinical entities, among all patients with unexplained recurrent fever episodes (16).

The medical approach in the daily clinical practice is aimed at evaluating all elements capable of explaining a systemic inflammatory condition in patients with not-otherwise explained recurrent fever episodes. In consideration of this, we have identified 54 patients with axial SpA during the past two years. None of them suffered from other known causes of fever at the time of the first assessment, while history recording, and physical examination corroborated the suspicion of SpA. A subsequent magnetic resonance of the sacroiliac joints highlighted the presence of radiologic signs consistent with an inflammatory skeletal involvement, with patients fulfilling the ASAS criteria for axial SpA (15).

Traditionally, axial SpA may be associated to extra-articular inflammatory manifestations, especially uveitis, inflammatory bowel diseases, and psoriasis (8). Conversely, despite episodes of recurrent fever have already been described in patients affected with SpA, fever does not figure among the common extra-articular SpA manifestations (6, 7, 31–33). Interestingly, Byun et al. have described 26 adult patients with SpA also suffering from recurrent fever episodes (7). Similarly, when comparing Behçet’s disease patients with SpA patients to investigate the presence of fever as a clinical feature of these conditions, Seyahi et al. observed a history of recurrent fever episodes in 8 out of 100 SpA patients (6). Despite these preliminary clues, it is not clear whether recurrent fever episodes should be considered as an expression of extra-articular inflammatory involvement or whether the axial SpA could have been part of a larger pathological picture. Based on a relatively wide cohort of patients from the AIDA Network USAID registry, we have described the clinical, laboratory and therapeutic features of these patients (16).

Except for uveitis, the frequency of classical extra-articular manifestations was quite similar to what observed in the literature among SpA patients. In particular, in the present cohort psoriasis was encountered in 9.3% of cases and inflammatory bowel diseases in 5.5%; these percentages overlap with those reported in non-febrile axial SpA (4%–9% and 5.5%–13% of cases for psoriasis and inflammatory bowel diseases, respectively). On the contrary, inflammatory ocular diseases were observed in 13% of febrile patients, which is quite lower than generally observed in the literature (from 22 to 37% of cases) (34).

Arthralgia, myalgia and peripheral arthritis accounted for the most frequent clinical manifestations associated to fever, while headache, diarrhea, abdominal pain and skin rash also occurred with a considerable frequency, as reported in Tables 2, 3 provides absolute and percentage frequencies of clinical and laboratory items included in ASAS criteria. In detail, the skin lesions observed consisted of erythematous, maculo-papular, urticarial rash, and pustular lesions. Noteworthily, several triggers have been reported to induce disease flares in about one third of patients, especially after psychological stress, intense physical activity, menstrual period, generalised cold exposure, and exposure to warm temperatures; 1 patient relapsed after SARS-CoV-2 vaccination.

**Table 3:** Frequency of the specific clinical and laboratory items included in the Assessment of SpondyloArthritis international Society (ASAS) criteria in the cohort of patients enrolled in the present study.

Clinical items of ASAS criteria	Number of patients (%)
Inflammatory back pain	54 (100)
Arthritis	22 (40.7)
Enthesitis (heel)	4 (7.4)
Uveitis	7 (13)
Dactylitis	4 (7.4)
Psoriasis	5 (9.3)
Chron’s/colitis	3 (5.5)
Good response to NSAIDs	24 (44.4)^a^
Family history for SpA	8 (14.8)
Elevated C reactive protein	30 (55.6)^b^
HLA-B27 positivity	5 (9.3)

HLA, human leukocyte antigen; NSAIDs, non-steroidal anti-inflammatory drugs; SpA: spondyloarthritis.

a All the 24 patients treated with NSAIDs during their history experienced at least a partial improvement in the low back pain.

b C reactive protein assessed outside fever bouts.

Whilst HLA-B27 is found in up to 90% of patients with SpA, the frequency of this haplotype was unusually low in our cohort (35). In addition, none of the patients enrolled in this study showed to suffer from radiographic ax-SpA. This evidence seems to suggest that patients with ax-SpA and fever episodes could differ from “classical” ax-SpA patients in the pathogenic aspects of musculoskeletal disease and in the extent of radiographic progression. This hypothesis is also strengthened by the low prevalence of HLA-B27 haplotype in our cases. These finding hints that other co-factors may play a major role in inducing this cluster of ax-SpA patients.

In over half of the cases, laboratory inflammatory markers showed to be increased before starting cDMARDs and/or bDMARDs. However, the concomitant use of corticosteroids in about 57% of patients could have reduced this frequency along with the baseline ESR and CRP values, which were substantially lower when compared to that reported by Byun et al. (7). Unfortunately, part of data collection was retrospective and did not allow an assessment of laboratory inflammatory markers during a fever attack in an adequate number of patients; consequently, this information was not statistically analysed.

Unlike findings reported by Byun et al. (7), a remarkable number of patients was treated with other than NSAIDs and glucocorticoids in this study. In particular, twenty-eight patients were treated with cDMARDs, while TNF antagonists represented the more frequently employed biotechnological treatment approach. Moreover, 11 patients have undergone anti-IL-1 treatment and 2 had been treated with the small molecule tofacitinib.

Anti-TNF agents appeared to play an important role in controlling febrile episodes and the other associated clinical inflammatory manifestations, allowing a significant glucocorticoid sparing effect. Most of the patients treated with TNF inhibitors benefited from at least a partial response, and more than one third of patients showed a complete response. A fair improvement was also observed in the clinimetry of the associated axial SpA. However, statistical significance was not reached, and this was probably due to the very short-term follow-up, corresponding to a median duration of 3 months. Along with clinical improvement, laboratory inflammatory markers showed a notable decrease. In particular, ESR decreased in a statistically significant manner, while the decrease of CRP bordered on statistical significance. The frequency of patients with increased inflammatory markers reduced without reaching statistical significance. This is probably due the relatively low values observed in the inflammatory markers at the start of treatment, as consequence of the concomitant glucocorticoids use.

Although recurrent fever episodes switch thought to autoinflammatory diseases, which are commonly treated with anti-IL-1 agents, the response to anakinra and canakinumab appeared less brilliant in the present cohort of patients. In particular, most of cases treated with IL-1 antagonists underwent a treatment failure and only a reduced number of patients benefited from a complete response. Noteworthily, when analysing the use of TNF inhibitors and IL-1 antagonists, a higher frequency of concomitant cDMARDs could be observed in patients treated with anti-TNF-agents. This could have favoured the therapeutic role of TNF inhibitors or have affected the response to anti-IL-1 agents. Regarding axial SpA activity during anti-IL-1 treatment, unfortunately BASFI and BASDAI were not collected during anakinra and canakinumab administration, as IL-1 antagonists are currently considered ineffective in axial SpA patients. It would have been interesting to look into this aspect, which should be the subject of further studies.

The use of colchicine and other cDMARDs as monotherapy led to a partial and complete response in a reduced number of patients. However, combination therapy of cDMARDs and bDMARDs showed a therapeutic role in increasing the frequency of complete and partial response, as observed in Figure 2.

Currently, we cannot provide a definitive conclusion about the pathogenetic nature of fever in these patients, but it may be assumed that fever represents an extra-articular, currently underestimated manifestation of SpA. Alternatively, we could address with a specific subgroup of axial SpA related to a primary involvement of innate immunity (11). Actually, whilst the reduced frequency of HLA-B27 allele positivity and the low frequency of uveitis seem to suggest this is a different clinical entity from the classical SpA, the pronounced response to anti-TNF therapy and the poor response to IL-1 inhibitors make this condition more similar to SpA than to a typical autoinflammatory conditions (36). The third hypothesis for which recurrent fever episodes can combine with axial SpA by a pure chance seems to be made unlikely by the relative high number of patients enrolled and by the good response obtained on both fever and musculoskeletal involvement with anti-TNF therapy. However, future research efforts with basic and clinical studies will have to clarify all these aspects.

Although the diagnostic delay of arthritic conditions is shrinking with the passing of decades and the increased awareness among physicians and patients, SpA is still widely under-recognized especially when extra-articular manifestations account for the presenting symptom (37–41). This seems especially true in axial SpA patients with fever, as previously observed by Byun et al., who reported a remarkable diagnostic delay and a lower chance to see a rheumatologist in early stage in such patients (7). In support of this, the diagnostic delay was about ten years in our cohort of patients, which is higher than generally observed in axial SpA (37, 39). Despite the wide diagnostic delay and the presence of generally negative prognostic factors toward radiographic progression, especially increased CRP, patients were diagnosed with non-radiographic axial SpA in all cases (42). This could be explained in different ways: we do not know the behavior of axial SpA associated with fever in the long-term. In addition, we do not know whether axial SpA and fever episodes started together or in different moments.

The major limitation of the present study is its pure clinical nature; it would be useful to investigate the issue of fever in axial SpA through laboratory studies. However, this work sheds new light on demographic, clinical and therapeutic features on the quite under-recognized subgroup of patients with recurrent fever episodes also suffering from axial SpA.

In conclusion, signs and symptoms referring to SpA should be inquired in patients with apparently unexplained recurrent fever episodes. The specific treatment for axial SpA may lead to a remarkable improvement in the frequency of fever episodes and in the control of concomitant inflammatory manifestations. Whether SpA is part of an already unrecognised systemic entity or fever is an underestimated extra-articular manifestation of SpA is subject of debate at current and requires further clinical and laboratory research.

## Data availability statement

The raw data supporting the conclusions of this article will be made available by the authors, without undue reservation.

## Ethics statement

The studies involving human participants were reviewed and approved by Azienda Ospedaliero Universitaria senese. Written informed consent to participate in this study was provided by the participants’ legal guardian/next of kin.

## Author contributions

AV and VC wrote the first draft of the manuscript and conceived, and designed the study. BF, IS, DO, RF, PR, FC, JS, CG, HG, CF, and PF were included based on the number of patients recruited in the AIDA network registry for USAIDs by February1st, 2022. IV, AT, GL, IA, JH-R, EW-S, VS, GR, MS, and FI were included in the authorship as investigators from the top contributor centers for any of the other AIDA network registries. AB was the bioengineer involved in the technical management of the platform and registries. DR edited and supervised the manuscript. BF and LC took care of the final revision of the manuscript. LC accounted for AIDA registries coordinator. All authors contributed to the article and approved the submitted version.

## Conflict of interest

The authors declare that the research was conducted in the absence of any commercial or financial relationships that could be construed as a potential conflict of interest.

## Publisher’s note

All claims expressed in this article are solely those of the authors and do not necessarily represent those of their affiliated organizations, or those of the publisher, the editors and the reviewers. Any product that may be evaluated in this article, or claim that may be made by its manufacturer, is not guaranteed or endorsed by the publisher.
